# A Pilot of Digital Whiteboards for Improving Patient Satisfaction in the Emergency Department: Nonrandomized Controlled Trial

**DOI:** 10.2196/44725

**Published:** 2023-03-21

**Authors:** Andrew D A Marshall, Mohammad Adrian Hasdianda, Steven Miyawaki, Guruprasad D Jambaulikar, Chenze Cao, Paul Chen, Christopher W Baugh, Haipeng Zhang, Jonathan McCabe, Lee Steinbach, Scott King, Jason Friedman, Jennifer Su, Adam B Landman, Peter Ray Chai

**Affiliations:** 1 Department of Emergency Medicine Harvard Medical School Boston, MA United States; 2 Department of Emergency Medicine Brigham and Women's Hospital Boston, MA United States; 3 Brigham Digital Innovation Hub Brigham and Women's Hospital Boston, MA United States; 4 Department of Psychosocial Oncology and Palliative Care Dana Farber Cancer Institute Boston, MA United States; 5 eVideon Coropration Grand Rapids, MI United States; 6 E Ink Corporation Billerica, MA United States; 7 Mass General Brigham Digital Somerville, MA United States; 8 The Koch Institute for Integrated Cancer Research Massachusetts Institute of Technology Cambridge, MA United States; 9 The Fenway Institute Boston, MA United States

**Keywords:** digital whiteboard, digital intervention, health technology, electronic paper, E-paper, patient satisfaction, communication, emergency department experience, experience, emergency, hospital, satisfaction, patient facing software, infrastructure, patient care, emergency department, patient communication, hospital setting, patient experience, perception, match pair, statistics, user experience, learning, Media and Technology Usage and Attitudes Scale, MTUAS, user interface, interface, design, Likert, Wilcoxon rank, Wilcox, exploratory data analysis, EDA, x^2, x^2^

## Abstract

**Background:**

Electronic paper (E-paper) screens use electrophoretic ink to provide paper-like low-power displays with advanced networking capabilities that may potentially serve as an alternative to traditional whiteboards and television display screens in hospital settings. E-paper may be leveraged in the emergency department (ED) to facilitate communication. Providing ED patient status updates on E-paper screens could improve patient satisfaction and overall experience and provide more equitable access to their health information.

**Objective:**

We aimed to pilot a patient-facing digital whiteboard using E-paper to display relevant orienting and clinical information in real time to ED patients. We also sought to assess patients’ satisfaction after our intervention and understand our patients’ overall perception of the impact of the digital whiteboards on their stay.

**Methods:**

We deployed a 41-inch E-paper digital whiteboard in 4 rooms in an urban, tertiary care, and academic ED and enrolled 110 patients to understand and evaluate their experience. Participants completed a modified Hospital Consumer Assessment of Health Care Provider and Systems satisfaction questionnaire about their ED stay. We compared responses to a matched control group of patients triaged to ED rooms without digital whiteboards. We designed the digital whiteboard based on iterative feedback from various departmental stakeholders. After establishing IT infrastructure to support the project, we enrolled patients on a convenience basis into a control and an intervention (digital whiteboard) group. Enrollees were given a baseline survey to evaluate their comfort with technology and an exit survey to evaluate their opinions of the digital whiteboard and overall ED satisfaction. Statistical analysis was performed to compare baseline characteristics as well as satisfaction.

**Results:**

After the successful prototyping and implementation of 4 digital whiteboards, we screened 471 patients for inclusion. We enrolled 110 patients, and 50 patients in each group (control and intervention) completed the study protocol. Age, gender, and racial and ethnic composition were similar between groups. We saw significant increases in satisfaction on postvisit surveys when patients were asked about communication regarding delays (*P*=.03) and what to do after discharge (*P*=.02). We found that patients in the intervention group were more likely to recommend the facility to family and friends (*P*=.04). Additionally, 96% (48/50) stated that they preferred a room with a digital whiteboard, and 70% (35/50) found the intervention “quite a bit” or “extremely” helpful in understanding their ED stay.

**Conclusions:**

Digital whiteboards are a feasible and acceptable method of displaying patient-facing data in the ED. Our pilot suggested that E-paper screens coupled with relevant, real-time clinical data and packaged together as a digital whiteboard may positively impact patient satisfaction and the perception of the facility during ED visits. Further study is needed to fully understand the impact on patient satisfaction and experience.

**Trial Registration:**

ClinicalTrials.gov NCT04497922; https://clinicaltrials.gov/ct2/show/NCT04497922

## Introduction

### Background

Electronic paper (E-paper), a display medium commonly found in devices such as Kindle e-readers, uses charged microcapsules to generate screen images. E-paper screens may have significant advantages in power saving when compared to light-emitting diode (LED) and liquid crystal display (LCD) monitors. They have no power requirement once set and will maintain the information displayed even during a power outage. E-paper displays, which can be similar in cost to high-end LCD monitors, have shown promise for use in health care settings [1]. When planning for use in the hospital, E-paper screens, which are not backlit, can also help limit the light pollution that can disturb patients at rest. They are ideal for displaying frequently updated information to patients without disrupting their clinical care.

In 2001, a report by the Institute of Medicine suggested the use of whiteboards to promote patient-centered care and improve communication [2]. Since then, the widespread use of whiteboards in clinical settings have produced evidence that whiteboards increase patient engagement, improve satisfaction, and reduce medical errors [3-5]. In inpatient units, implementing a daily routine to update whiteboards has had moderate success in providing near–real-time information about daily plans for patients [5]. The nature of emergency care makes the emergency department (ED) one of the most information-intensive areas of the hospital [6]. High throughput, high acuity, and complex care coordination have created a need for whiteboards to augment the efficiency of clinical staff [7]. However, systems for consistently and accurately maintaining patient-facing whiteboards and the effect on patient experience in the ED have not been well explored.

### Study Rationale

The COVID-19 pandemic has created significant barriers to communication within health care settings [8]. The need for masking and social distancing can inhibit communication by decreasing in-person updates between clinicians and patients [9]. Additionally, limits placed on the number of family members allowed in the hospital may hamper traditional methods in which clinicians could discuss hospital course and disposition plans. These factors coupled with the typical lag time it takes for a patient to complete an ED visit can leave patients in a state of uncertainty. This can decrease patient satisfaction and blunt patient engagement in their care [10]. Previous research has demonstrated that patients, when adequately engaged via a patient-centered model of care, have increased satisfaction and better outcomes [11]. In the ED, realistic estimations of wait times, strong interpersonal communication, and effective information delivery improve satisfaction [12,13]. With the decline of in-person interactions guided by social distancing and with communication further hindered by mask wearing, the ability to provide key information in a chaotic environment such as the ED may improve patient satisfaction and engagement. In other care settings, patient portals have shown promise in improving patient engagement [11,14]. The use of patient portals by ED patients is limited at baseline [15], despite patients comfort with the use of digital health technology [16]. Black patients, Hispanic patients, and older patients disproportionately use the ED for care [17,18]. These patients have been found to be underrepresented in their engagement with patient portals [15]. Recent emphasis by the Office of the National Coordinator for Health Information Technology has focused on giving patients more control and access to their medical record [19]. To achieve these goals within health care, inclusive digital solutions must be considered for underserved patient populations [20].

To improve communication, information accuracy, reduce administrative burden, and mitigate concerns about infectious exposure, digital whiteboards are a logical next step to replace traditional whiteboards in ED and other care settings.

### Specific Objectives

**Figure 1:**
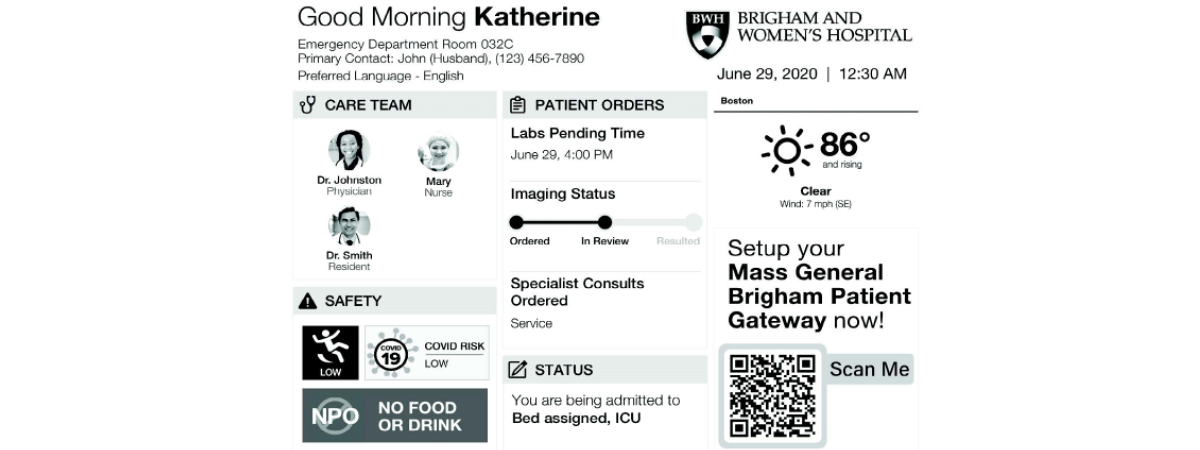
Digital whiteboard display. Orienting information, care team information, safety information, patient orders, and disposition information were selected for display. Additional information regarding enrolment in Patient Gateway, our health system’s patient portal, was included to further drive patient engagement. ICU: intensive care unit; NPO: nil per os (nothing by mouth).

Although E-paper has been used in hospitals and commercially as digital signage, its applications for patient engagement have not been studied. In this investigation, we aimed to deploy a patient-facing E-paper digital whiteboard (Figure 1) to provide key ED-related information pertaining to the patient’s ED course. We sought to assess patients’ satisfaction with their ED stay after relevant orienting and clinical information was displayed in real time. Lastly, we aimed to understand our patients’ overall perception of the impact of the digital whiteboards on their stay.

## Methods

### Study Design

Since E-Paper and patient-facing digital whiteboards have not been previously studied in the ED setting, we decided to conduct a case-control study to understand the potential effect of the E-paper digital whiteboard on satisfaction. We considered a single-arm pilot trial but wanted to establish a control group to better understand the baseline satisfaction and attitudes toward technology of ED patients. Limited study resources made it impractical to equip every ED room with a digital whiteboard, so we limited our study to 4 ED rooms for our intervention group. A true “randomized control trial,” while ideal, was not feasible.

### Sample Size Calculation

Given the pilot nature of the study and the unclear potential effect size we would expect from the digital whiteboard platform, we did not calculate a sample size. This pilot study aimed to establish the baseline for satisfaction and explore trends toward significance to plan future interventions and studies.

### Digital Whiteboard Display

**Figure 2:**
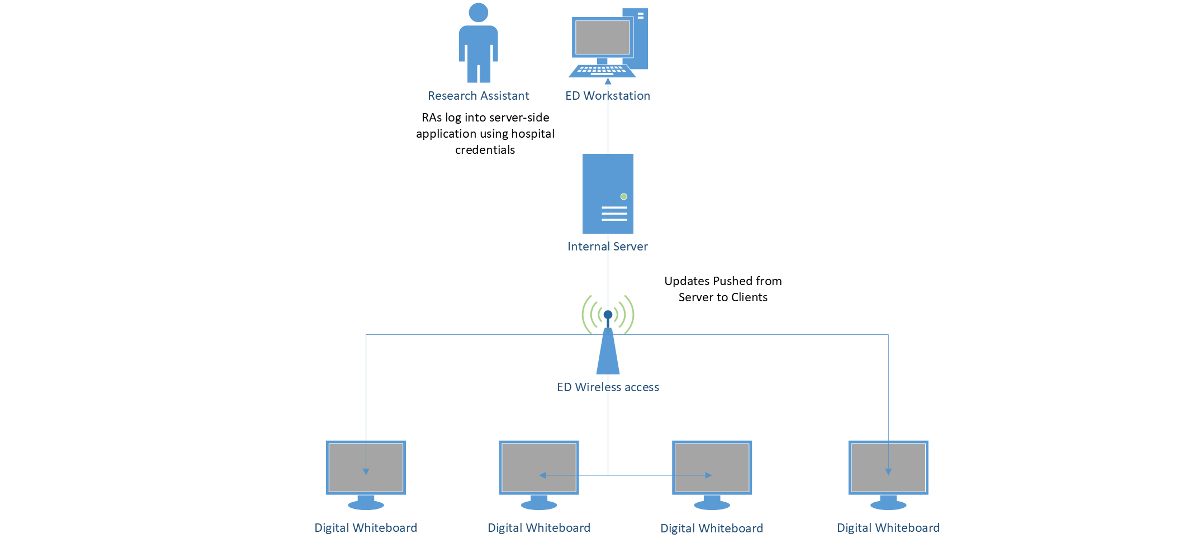
Information system setup. Personnel conducting the study were able to log-in and enter information into the eVideon secure server-side client using OAuth, with no exposure of hospital credentials or protected health information (PHI) to external vendor. ED: emergency department; RA: research assistant.

We used a 41-inch black-and-white (grey-scale) E-paper display (E Ink Corporation) with an Android operating system running the Insight digital whiteboard client as a digital whiteboard. Although color versions of E-paper screens are available [1], a black-and-white display is ideal for communicating basic information. The E-paper display can be powered via AC power or power over ethernet. We chose to use standard wireless network connection due to the cost of installing power over ethernet. We provisioned a secure server behind our enterprise firewall. We loaded a custom server-side application (eVideon Health) onto the internal server and a receiving application onto the client-side digital whiteboard. Our research team was able to securely log in using their hospital credentials, allowing us to enter data into customized fields to display on the E-paper whiteboard (Figure 2). We made updates every 30 minutes and pushed data from the server-side application to the client-side application.

In collaboration with ED nursing and physician leadership, we defined key features of the ED course that were perceived as important to keep patients updated over the course of their care. Through an agile sprint, we developed key data fields (care team, safety information, orders, and disposition information) for the digital whiteboard display and modified them according to formative feedback from the study team and ED leadership. We then installed E-paper whiteboards in 4 ED rooms using a mounting bracket and drywall screws. We strategically placed E-paper whiteboards where they could be easily viewed by patients in the room, yet not facing the entry to the patient room to prevent protected health information from being viewed by others walking through the ED. The 41-inch E-paper whiteboard was placed on an empty wall and did not displace any other hospital equipment.

### Setting and Participant Recruitment

We conducted a prospective cohort trial of patients over the age of 18 years presenting to the ED for care to understand the effect of our digital whiteboard on patient satisfaction. Our study site was a single urban, academic, and tertiary care hospital in Boston, Massachusetts, with an annual visit volume of approximately 63,000 patients. We enrolled participants on a convenience basis after triage into private rooms in the ED with a digital whiteboard. Nursing triage staff assigned patients to these rooms as part of their routine clinical practice. English-speaking patients with an expected ED stay of >2 hours were included in the study, regardless of chief complaint, race, age, or other factors. Non–English-speaking patients, patients who presented with a primary psychiatric complaint, patients boarding in the ED, patients at high risk for COVID-19 or with confirmed infection, or patients who were deemed too ill or unable to participate by their clinical team were excluded from the study.

Participants triaged to rooms without digital whiteboards comprised our control group. Patient experiences with digital whiteboards were compared to the standard of care in our ED at the time, which did not involve traditional whiteboards. Patients did have access to a patient portal linked to the electronic health record (EHR), which could also provide results in real time. Patients in the control group had routine updates provided to them at the discretion of the ED team, in line with the standard of care. Participants triaged to rooms with digital whiteboards comprised our intervention group. For participants involved in the study, these digital whiteboards displayed relevant clinical updated by a research assistant (RA).

### Data Collection

Once roomed, we approached participants to explain the study and obtain written consent. Baseline demographic information was reviewed in our EHR and recorded by an RA in REDCap (Research Electronic Data Capture; Vanderbilt University), a secure web-based software platform to support data capture for research studies [21,22]. To better understand and compare the baseline attitudes toward technology in our 2 study groups, we delivered the Media and Technology Usage and Attitudes Scale (MTUAS) [23] in our initial survey. The MTUAS questionnaire asked patients to rank their use and comfort with technology on a numerical scale.

To assess patient satisfaction, we issued a modified Hospital Consumer Assessment of Healthcare Providers and Systems (HCAHPS) [24] questionnaire. The survey administered was based on the standard questionnaire administered to ED patients in our academic center, which asked patients to rank their experiences in the ED on a 4-point Likert scale. This satisfaction survey was provided again by the RAs at the time of final disposition for all participants in the study.

To assess the perceptions of the impact of the digital whiteboards on their stay, patients in the intervention arm received a short quantitative survey. Patients in this arm were asked if they liked the digital whiteboard, if they had trouble resting, if they found it distracting, and if they felt that it helped to understand their ED stay. They were also surveyed about their preference for a room with a digital whiteboard and about their perception of the size of the whiteboard.

**Figure 3:**
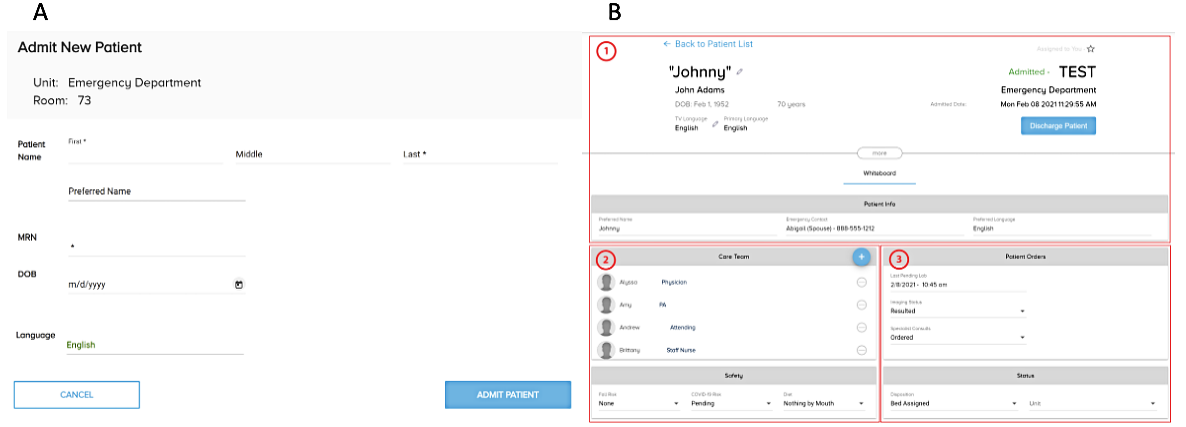
Research assistant data entry interface. (A) User interface designed and implemented by eVideon corporation that allowed for entry and updates on individual whiteboards. (B) In section 1, basic patient information (name, DOB, and language) was manually input via free-text fields from data selected from the electronic health record (EHR). Date and time information was generated automatically. In section 2, care team information and safety information were selected from a prepopulated dropdown menu. In section 3, results for laboratory tests, imaging, and disposition from the emergency department were selected from dropdown menus and time stamped. DOB: date of birth; MRN: medical record number; PA: physician assistant.

Once a patient was enrolled, an RA logged into the research user interface and activated the digital whiteboard. Relevant clinical data were entered by an RA onto the whiteboard using the interface, illustrated in Figures 3, at 30-minute intervals. This information was inputted directly from the EHR. Any modifications made in the user interface were automatically pushed to the corresponding digital whiteboard screen in the appropriate room.

### Study Measures

#### HCAHPS Questionnaire

To evaluate patient satisfaction, we used a modified HCAHPS questionnaire that was delivered to the patient on paper by an RA before departure from the ED. These questionnaires evaluate satisfaction in nursing communication, provider communication, responsiveness of hospital staff, discharge information, and overall hospital experience. We removed the open-ended questions from our standard Department of Emergency Medicine HCAHPS survey, limiting questions to only those that could be answered with a 4-point Likert scale (“No”; “Yes, somewhat”; “Yes, mostly”; and “Yes, definitely”). We assigned 1 point for “No” or up to 4 points for “Yes, definitely.” We asked a single question that was ranked on a numerical scale (1-10) regarding the likelihood to recommend this facility to family and friends (Multimedia Appendix 1).

#### MTUAS Tool

To establish the baseline attitudes of our patient population to technology, we used the MTUAS tool. The MTUAS tool, designed to standardize the measurement of technology use, has strong reliability and validity [23]. Across several categories of technology use, including cell phone use, computer use, and social media use, patients were asked to rate their use and comfort with technology on a numerical scale (Multimedia Appendix 1).

#### Other Survey Components

For the intervention group, we developed and implemented a custom survey (Multimedia Appendix 2), using a 5-point scale (“Not at all,” “A little bit,” “Moderately,” “Quite a bit,” and “Extremely”) and “Yes” and “No” questions, to assess the opinions of patients regarding the digital whiteboard. Patients were also permitted to provide qualitative, free-text feedback.

### Statistical Analysis

For our pilot study, we performed an exploratory data analysis. Data were visualized and analyzed for distribution and skew before a statistical test was chosen. We completed data analysis using R statistical software (version 4.1.2; R Foundation for Statistical Computing).

#### Demographic Comparison

For descriptive demographic variables, we calculated means and SDs. Based on previous data that suggest a nonnormal distribution of age in those presenting to the ED [18], we opted to use the Wilcoxon rank sum test. We compared sex between the 2 cohorts using a 2-sided *t* test. We compared the distribution of the categorical variables, race, and ethnicity using the chi-square test of independence.

#### MTUAS Scores

We calculated mean MTUAS scores and SDs for each group aggregated by the following major categories: phone ownership, computer ownership, texting, calling, smartphone use, email, attitude toward technology, and anxiety without technology. Data exploration of each category showed a nonnormal distribution with left skew. We compared each category between 2 groups using the Wilcoxon rank sum test.

#### HCAHPS Scores

To evaluate changes in patient satisfaction, which were also left skewed, we compared the 2 groups with a Wilcoxon rank sum test.

#### Other Survey Components

The results of the custom survey were reported in the form of absolute numbers and percentages. No further analysis was performed.

### Ethics Approval

This study was reviewed and approved by the Mass General Brigham Institutional Review Board (2020P002382) and registered on ClinicalTrials.gov (NCT04497922). Written consent was obtained by RAs in the ED (Multimedia Appendix 3). Research data were deidentified and anonymously stored in a REDCap database. Subjects were not compensated for their participation in this study.

## Results

### Participants

**Figure 4:**
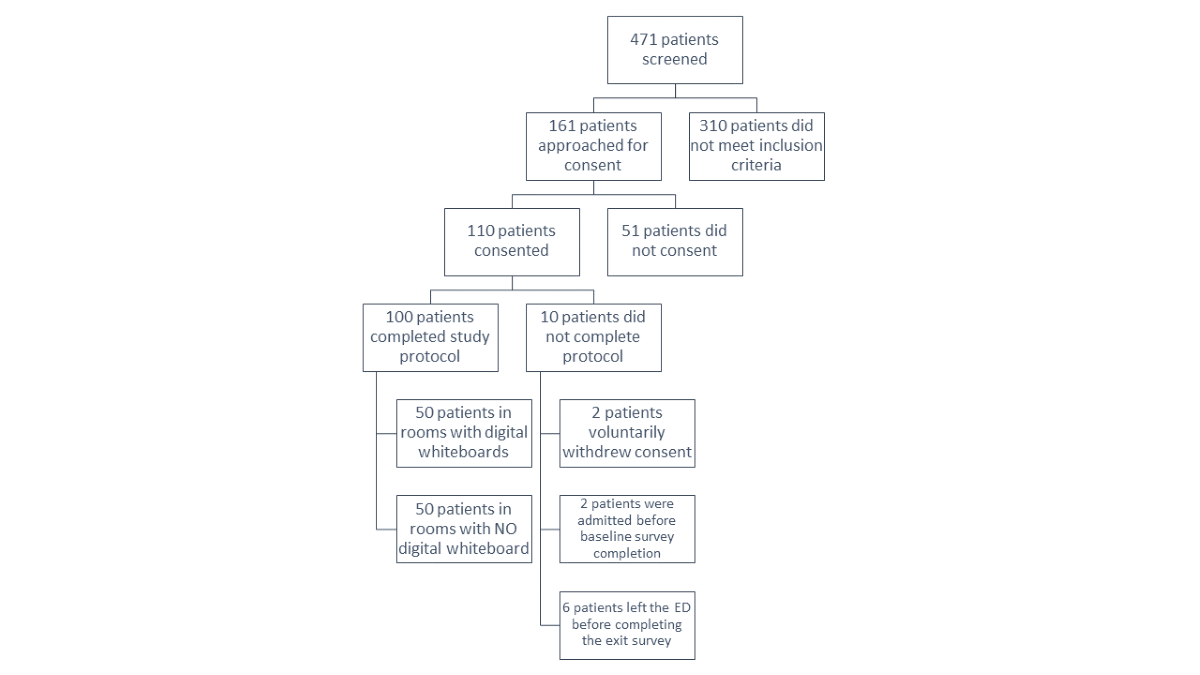
Study flow diagram. ED: emergency department.

Over the study period, we screened 471 participants. Of the 471 screened participants, we approached 161 participants, of whom 110 provided verbal consent. We enrolled 56 participants in the E-paper cohort, whereas 54 participants were enrolled in the control cohort. Two participants voluntarily withdrew consent and did not complete the final assessment. Two participants were admitted to the hospital prior to the initiation of the baseline assessment. Six participants were discharged, admitted, or left the ED against medical advice prior to the completion of the final assessment. The demographics of the participants that did not complete the final assessment are similar and are documented in Multimedia Appendix 4. A total of 100 consenting participants completed all measures associated with the study (Figure 4).

### Baseline Characteristics

The control and intervention groups were similar in all baseline characteristics (Table 1). Mean age was 45 (SD 17.5) years in the control group and 50 (SD 18.6) years in the intervention group. In both cohorts, most participants were female (control: 38/50, 76%; intervention: 30/50, 60%) and White (control: 32/50, 64%; intervention: 29/50, 58%). Other demographic groups including Black, Asian, Hispanic, and “Other” patients were similarly represented. As detailed in Table 2, participants in both groups had a general positive attitude toward the use of technology, and most participants owned both a phone and a computer. Participants reported using digital devices for texting, calling, and emailing. Patients lost to follow-up also had similar baseline characteristics.

**Table 1:** Group demographics.

Demographics			Control (n=50)		Intervention (n=50)		*P* value
Age (years), mean (SD)			45.16 (17.5)		50.60 (18.6)		.16
Female, n (%)			38 (76)		30 (60)		.09
**Race,** **n (%)**							.28
	Asian	1 (2)		5 (10)			
	Black or African American	10 (20)		12 (24)			
	Other	7 (14)		4 (8)			
	White	32 (64)		29 (58)			
**Ethnicity,** **n (%)**							.41
	Hispanic	7 (14)		3 (6)			
	Non-Hispanic	41 (42)		45 (90)			
	Unavailable	2 (4)		2 (4)			

**Table 2:** Attitudes toward technology (Media and Technology Usage and Attitudes Scale results).

Item			Control (n=50)		Intervention (n=50)		*P* value
**Device ownership, n (%)**							
	Smartphone	49 (98)		44 (88)		.08	
	Computer or laptop	40 (80)		33 (66)		.11	
**Usage subscales (1-10)^a^, mean (SD)**							
	Texting	7.12 (1.61)		7.61 (2.14)		.08	
	Calling	7.38 (1.61)		7.27 (2.08)		.82	
	Smartphone	6.31 (1.97)		6.43 (2.54)		.76	
	Email	5.37 (2.48)		6.22 (2.65)		.14	
**Attitudes subscales (1-5)^b^, mean (SD)**							
	Positive attitude	3.76 (.720)		3.88 (.591)		.44	
	Anxiety without technology	3.00 (.673)		3.18 (.647)		.35	

^a^1=Never, 2=Once a month, 3, Several times a month, 4=Once a week, 5=Several times a week, 6=Once a day, 7=Several times a day, 8=Once an hour, 9=Several times an hour, and 10=all the time.

^b^1=Strongly disagree, 2=Disagree, 3=Neither agree nor disagree, 4=Agree, and 5=Strongly agree.

### Patient Satisfaction

**Figure 5:**
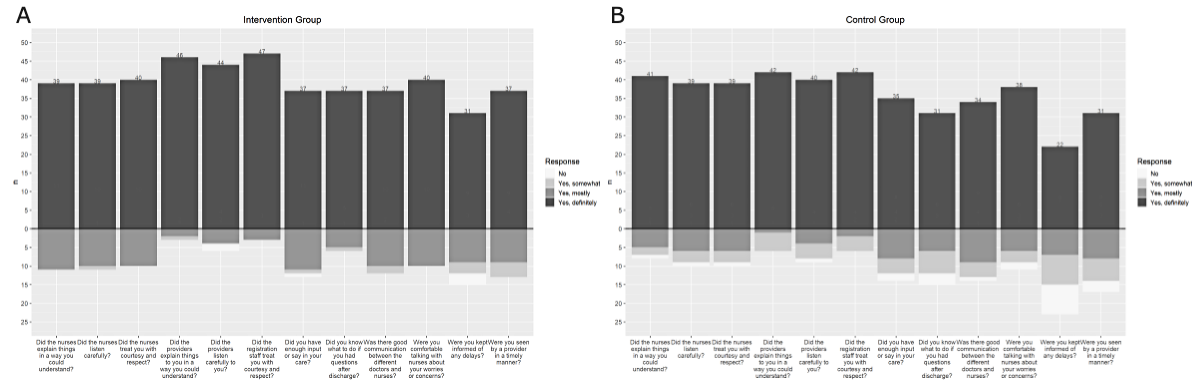
Distribution of Hospital Consumer Assessment of Health Care Provider and Systems (HCAPHS) scores: (A) intervention and (B) control.

At baseline, patients had high satisfaction with their ED visit. The median score for the control group for each question was 3 (“Yes, definitely”; Figure 5A). This was also true of the intervention group (Figure 5B). However, significantly more patients in the intervention group answered “Yes, definitely” when asked, “Were you kept informed about any delays?” (*P*=.03) and “Did you know what to do if you had questions/concerns after discharge?” (*P*=.02; Figure 5). The *P* values of these groups, compared by Wilcoxon rank sum, are recorded in Table 3. There was a significant difference in the 2 groups when asked, “How likely would you be to recommend this facility to your family and friends?”; the control group reported an average score of 9.22 and the intervention group reported a higher average score of 9.66 (*P*=.045).

**Table 3:** Significance of postvisit Hospital Consumer Assessment of Health Care Provider and Systems score in control group versus intervention group.

Question	Satisfaction score, mean (SD)		*P* value
	Control	Intervention	
Were you seen by a care provider in a timely manner?^a^	2.39 (.939)	2.66 (.626)	.21
Were you kept informed about any delays?^a^	1.96 (1.19)	2.48 (.888)	.03
Were you comfortable talking with nurses about your worries or concerns?^a^	2.63 (.782)	2.80 (.404)	.59
Was there good communication between the different doctors and nurses?^a^	2.58 (.739)	2.71 (.540)	.51
Did you know what to do if you had questions/concerns after discharge?^a^	2.41 (.956)	2.83 (.433)	.02
Did you have enough input or say in your care?^a^	2.55 (.818)	2.68 (.621)	.62
At the time of your arrival, did the registration staff treat you with courtesy and respect?^a^	2.79 (.582)	2.94 (.239)	.24
Did the care providers listen carefully to you?^a^	2.69 (.713)	2.80 (.639)	.37
Did the care providers explain things in a way you could understand?^a^	2.77 (.627)	2.91 (.344)	.26
Did nurses listen carefully to you?^a^	2.69 (.683)	2.76 (.476)	.99
Did nurses treat you with courtesy and respect?^a^	2.69 (.683)	2.80 (.404)	.81
Did nurses explain things in a way you could understand?^a^	2.75 (.630)	2.78 (.418)	.59
How likely are you to recommend this facility to family or friends?^b^	9.22 (1.65)	9.66 (1.24)	.045

^a^0=No, 1=Once a month, 2=Several times a month, and 3=Once a week.

^b^From 1=Least likely to 10=Most likely.

### Patient Perceptions of Digital Whiteboards

We sought feedback on the whiteboards from our intervention group (Table 4). Nearly all participants (48/50, 96%) in this group reported that they preferred a room with a whiteboard. The entire group of participants agreed that the size of the whiteboard was an adequate size to communicate information appropriately. Additionally, 84% (n=42) of those in the intervention group reported that they liked the digital whiteboard “Quite a bit” or “Extremely.” They also reported that the screen was not distracting (n=46, 92%). Only 2 (4%) of these participants reported that they had trouble resting with the screen in the room. Lastly, 70% (n=35) of participants surveyed reported that the digital whiteboard helped them understand their ED stay “Quite a bit” or “Extremely.”

**Table 4:** Quantitative assessment of perceptions of digital whiteboard (intervention group).

Question and response		Participant (n=50), n (%)
**Did you like the digital whiteboard?**		
	Extremely	22 (44)
	Quite a bit	20 (40)
	Moderately	4 (8)
	A little bit	4 (8)
	Not at all	0 (0)
**Preference for a room with a digital whiteboard?**		
	Yes	48 (96)
	No	2 (4)
**Did you have trouble resting with the screen?**		
	No	48 (96)
	Yes	2 (4)
**Was the digital whiteboard distracting?**		
	Not at all	46 (92)
	A little bit	2 (4)
	Moderately	1 (2)
	Quite a bit	1 (2)
**Was the digital whiteboard adequately sized?**		
	Yes	50 (100)
	No	0 (0)
**Did the digital whiteboard help you understand your ED^a^ stay?**		
	Extremely	20 (40)
	Quite a bit	15 (30)
	Moderately	5 (10)
	A little bit	7 (14)
	Not at all	3 (6)

^a^ED: emergency department.

### Unintended Outcomes

There were no recorded unintended outcomes from our study. Anecdotally, we did not have any reports of increased interruptions or reports of increased anxiety or impatience from patients who received additional information regarding their care.

## Discussion

### Principal Findings

In this investigation, we demonstrated the feasibility of the use of an E-paper digital whiteboard as a tool for communication in the ED. We sought to replace much of the work required to update a whiteboard by providing a simulation of automation of these processes while providing an unobtrusive screen and viewing experience using E-paper. In this formative work, we were able to leverage an open Android operating system to develop a back-end, Health Insurance Portability and Accountability Act–compliant interface that can be updated and pushed in real time to patients. Even with updates every 30 minutes in this investigation, participants felt an impact on their ED stay. With future iterations of the technology, integration with the EHR can automate and provide real-time updates of key hospital events such as laboratory results, transit times, plans for imaging and other testing, and progress of consults to better inform patients of their hospital course.

This intervention has the potential to influence satisfaction in ED patients. The display of our selected features resulted in significant differences in patient satisfaction between the control and intervention group. Patients also had a generally positive perception of the digital whiteboard, and a majority felt as though it played some role in helping them understand their ED stay. Patients in the intervention group had significantly higher scores when asked if they were kept informed about delays and if they knew what to do if they had questions after discharge. They were also significantly more likely to recommend the facility to their family and friends. This suggests that timely information improves the comprehension of the ED stay and could contribute to the adherence and effectiveness of post-ED discharge instructions. This and similar interventions may positively influence the overall perceptions of the hospital facility.

### Comparison With Prior Studies

Our findings are consistent with previous research that shows that the actual length of wait times are often not as important as realistic communication regarding wait times [12]. The implications of these data are that the deployment of E-paper digital display screens may impact the patient experience and could help address communication challenges in the ED.

Although whiteboards have become commonplace in inpatient units in the hospital as a method for clinical care teams to communicate expected plans of care and key contact information for patients, their actual use is often suboptimal [25]. The traditional dry erase whiteboard requires staff to constantly update tasks within the board, which frequently can be missed in the setting of a busy hospital and clinicians managing increasing volumes of patients.

Our study demonstrated that patients were more satisfied in their overall ED stay and more likely to recommend the hospital to friends and family when provided near–real-time updates about key features of their visit via digital whiteboard. Patients that receive adequate communication about delays in the ED have increased satisfaction [12], which can lead to better engagement with care [11]. With many hospital EDs to choose from in a busy urban area, even small changes in satisfaction may impact hospital choice [26].

Our data should also be considered in the context of the COVID-19 pandemic. During this study, our ED experienced several waves of COVID-19 infection resulting in the enactment of several key policies to minimize in-person patient contact when possible as an infection control strategy. These included enabling phone-based triage of patients in ED rooms, restricting access to the ED for family and significant others, and strict rules surrounding face masks for all staff and patients. These policies may have inadvertently resulted in decreased time spent with patients by both physicians and nursing staff and may have adversely impacted patient satisfaction and outcomes [8,27]. Under these conditions, the provision of information on an unobtrusive but legible digital display screen could have improved patient perceptions of their emergency care.

### Future Directions

Although we have demonstrated the feasibility of digital display screens to provide patient information in the ED, future work should be done to facilitate integration with the EHR. This would enable real-time exchange of information such as the status of laboratory results, wait times for imaging studies, or the progress of consults, which typically are unknown to patients. These automated features may help patients anticipate their ED course and impact satisfaction. Although we only addressed roomed ED care, similar information could be displayed on portable devices to address patients receiving care in hallways or in other alternative care settings. One key demographic we did not enroll in our study was patients in our ED observation unit who are anticipated to require care up to 48 hours. It is likely that patients who are in observation status may benefit from updates over the course of a longer stay that may better inform them of common testing procedures and the timing for them. Given the increase in the use of observation units in EDs in the United States and the increased time patients spend in the ED due to crowding issues, future work should be done to understand the effectiveness of continuous updates during longer emergency stays. Future work should also seek to provide translated display screens to understand the effectiveness of these systems in non–English-speaking patients. We often fail to accurately characterize satisfaction and understanding within this group of patients, and a language-appropriate digital intervention may make a larger difference in this group and serve to promote equitable health care communication. Finally, iterative refinement of data elements and future patient- and provider-focused work around defining other features of E-paper displays that may impact the patient experience in the ED should be investigated.

### Limitations

Our study had several limitations. First, we conducted this small pilot study at a large, single-site, and academic ED. Resources and the feasibility of using digital whiteboards screens may vary at other institutions. Second, our study was limited by size. Patients generally had a positive view of their ED stay, providers, and nurses at baseline. Changes in these views might be small, and a larger sample size is necessary to avoid type II error. We did not calculate a sample size, and our pilot study may not be powered to detect significant changes, although our results point to certain potential effects. Because of the small size of our study, we were unable to assess the effects of this form of digital communication on racial and ethnic subgroups that might also experience emergency care differently. Third, due to the pilot nature of the study and the fact that we could only program the digital display screen in English, we only enrolled patients who could read and speak English. This excluded individuals who are non-English speaking. Language presents a significant barrier to communication, and non–English-speaking patients often experience this in the form of health care disparities. Lastly, our study simulated a truly automated process. Study personnel were required to manually input data, albeit remotely. This may have limited the effect of real-time information flow to the patient.

### Conclusion

E-paper is an acceptable and technologically viable medium for information exchange in the ED and may influence patient satisfaction. E-paper digital whiteboards have the potential to replace traditional whiteboards while being similar in cost and physical footprint to LCD screens, with added benefits for patient care and staff. They are easy to install, easy to read, and well accepted by ED patients. Despite the pilot nature of our study, we found that providing timely information surrounding key aspects of ED care improves satisfaction when patients were asked about delays and how to address concerns after discharge. We also found that it influences patients’ likelihood to recommend the facility. Future studies should include a larger group of patients, different care settings, and additional language support. For E-paper to be a viable operation solution in the hospital, automated integration with the EHR via push or pull from the device needs to be further delineated.

## Appendix

Multimedia Appendix 1 Baseline survey.

Multimedia Appendix 2 Exit survey.

Multimedia Appendix 3 Consent form.

Multimedia Appendix 4 Demographics of excluded patients.

## Abbreviations

E-paper: Electronic paper
ED: emergency department
EHR: electronic health record
HCAHPS: Hospital Consumer Assessment of Healthcare Provider and Systems
LCD: liquid crystal display
LED: light-emitting diode
MTUAS: Media and Technology Usage and Attitudes Scale
RA: research assistant
REDCap: Research Electronic Data Capture

## Data Availability

Data for this study have been made publicly available [28].
