# Supplementary material for: A Pilot of Digital Whiteboards for Improving Patient Satisfaction in the Emergency Department: Nonrandomized Controlled Trial
Source: JMIR Form Res. 2023 Mar 21;7:e44725. doi: 10.2196/44725 (PMC10131606; doi:10.2196/44725)
Supplement: Multimedia Appendix 1 [file formative_v7i1e44725_app1.pdf]

Appendix 1: Baseline survey

Thank you for agreeing to participate in this study.

The questions listed below review topics related to demographics, medical history, medication use, and perceptions and consequences related to your pain. Some of these questions may be of personal or sensitive nature. Although it is hoped that you will answer all questions in the survey, you may skip over any questions that you choose not to answer.

Part A: The Media and Technology Usage and Attitudes Scale (MTUAS) questions

1. Do you own a smartphone? Yes or No

2. Do you own a laptop, or desktop computer? Yes or No

* If no to question 1, complete sections 3 and 5
* If no to question 2, complete sections 4 and 5

For the following questions use the following 10 item frequency scale:
Never (1)
Once a month (2)
Several times a month (3) Once a week (4)
Several times a week (5) Once a day (6)
Several times a day (7) Once an hour (8)
Several times an hour (9) All the time (10)

3. Please indicate how often you do the following email activities on your laptop or desktop?
    1. Send, receive and read e-mails: 1 2 3 4 5 6 7 8 9 10
    2. Check your personal email: 1 2 3 4 5 6 7 8 9 10
    3. Check your work of school email: 1 2 3 4 5 6 7 8 9 10
    4. Send or receive files via email: 1 2 3 4 5 6 7 8 9 10

4. Please indicate how often you do each of the following activities on your mobile phone.
    1. Send and receive text messages on a mobile phone: 1 2 3 4 5 6 7 8 9 10
    2. Make and receive mobile phone calls: 1 2 3 4 5 6 7 8 9 10
    3. Check for text messages on a mobile phone: 1 2 3 4 5 6 7 8 9 10
    4. Check for voice calls on a mobile phone: 1 2 3 4 5 6 7 8 9 10
    5. Read email on a mobile phone: 1 2 3 4 5 6 7 8 9 10
    6. Get directions or use GPS on a mobile phone: 1 2 3 4 5 6 7 8 9 10
    7. Browse the web on a mobile phone: 1 2 3 4 5 6 7 8 9 10
    8. Listen to music on a mobile phone: 1 2 3 4 5 6 7 8 9 10
    9. Take pictures using a mobile phone: 1 2 3 4 5 6 7 8 9 10
    10. Check the news on a mobile phone: 1 2 3 4 5 6 7 8 9 10
    11. Record video on a mobile phone: 1 2 3 4 5 6 7 8 9 10
    12. Use apps (for any purpose) on a mobile phone: 1 2 3 4 5 6 7 8 9 10
    13. Search for information with a mobile phone: 1 2 3 4 5 6 7 8 9 10
    14. Use your mobile phone during class or work time: 1 2 3 4 5 6 7 8 9 10

5. For the next series of statements use the following scale:

Strongly Agree (5)
Agree (4)
Neither Agree nor Disagree (3) Disagree (2)
Strongly Disagree (1)

1. I feel it is important to find any information whenever I want online: 1 2 3 4 5
2. I feel it is important to be able to access the Internet any time I want: 1 2 3 4 5
3. I think it is important to keep up with the latest trends in technology: 1 2 3 4 5
4. I get anxious when I don’t have my cell phone: 1 2 3 4 5
5. I get anxious when I don’t have the internet available to me: 1 2 3 4 5
6. I am dependent on my technology: 1 2 3 4 5
7. Technology will provide solutions to many of our problems: 1 2 3 4 5
8. With technology, anything is possible: 1 2 3 4 5
9. I feel I get more accomplished because of technology: 1 2 3 4 5
10. New technology makes people waste too much time: 1 2 3 4 5
11. New technology makes life more complicated: 1 2 3 4 5
12. New technology makes people more isolated: 1 2 3 4 5

Part B: For the next series of statements use the following scale to describe your current stay in the emergency department (HCAHPS):

For the purpose of this survey, the term "care provider" includes physicians and physician assistants involved in your care.	
Were you seen by a care provider in a timely manner?	No, Yes somewhat, Yes mostly, Yes definitely
At the time of your arrival, did the registration staff treat you with courtesy and respect?	No, Yes somewhat, Yes mostly, Yes definitely
Were you kept informed about any delays?	No, Yes somewhat, Yes mostly, Yes definitely, Not applicable
Did the care providers listen carefully to you?	No, Yes somewhat, Yes mostly, Yes definitely
Did the care providers explain things in a way you could understand?	No, Yes somewhat, Yes mostly, Yes definitely
Did you know what to do if you had questions/concerns after discharge?	No, Yes somewhat, Yes mostly, Yes definitely, Not applicable

The next questions focus specifically on your experience with nurses during this visit.	
Did nurses treat you with courtesy and respect?	No, Yes somewhat, Yes mostly, Yes definitely
Did nurses listen carefully to you?	No, Yes somewhat, Yes mostly, Yes definitely
Did nurses explain things in a way you could understand?	No, Yes somewhat, Yes mostly, Yes definitely
Was there good communication between the different doctors and nurses?	No, Yes somewhat, Yes mostly, Yes definitely
Were you comfortable talking with nurses about your worries or concerns?	No, Yes somewhat, Yes mostly, Yes definitely
Did you have enough input or say in your care?	No, Yes somewhat, Yes mostly, Yes definitely

How likely would you be to recommend this facility to your family and friends?	Likely 0-10
