# Supplementary material for: A Pilot of Digital Whiteboards for Improving Patient Satisfaction in the Emergency Department: Nonrandomized Controlled Trial
Source: JMIR Form Res. 2023 Mar 21;7:e44725. doi: 10.2196/44725 (PMC10131606; doi:10.2196/44725)
Supplement: Multimedia Appendix 2 [file formative_v7i1e44725_app2.pdf]

Appendix 2: Exit survey

Thank you for agreeing to participate in this study.

The questions listed below review topics related to demographics, medical history, medication use, and perceptions and consequences related to your pain. Some of these questions may be of personal or sensitive nature. Although it is hoped that you will answer all questions in the survey, you may skip over any questions that you choose not to answer.

Part A: Perceptions of the Virtual Whiteboard (Skip these questions if you had a room without a virtual white board)

1. Did you like the virtual white board?

Not at all	A little bit	Moderately	Quite a bit	Extremely
0	1	2	3	4

2. When given the opportunity to have a room with a virtual white board versus a standard room without a virtual white board in the emergency department, what would you prefer?
    - o A room with a virtual whiteboard
    - o A room without a virtual whiteboard

3. Did you have trouble resting with the virtual white board?

Not at all	A little bit	Moderately	Quite a bit	Extremely
0	1	2	3	4

4. Was the virtual white board distracting you during your stay?

Not at all	A little bit	Moderately	Quite a bit	Extremely
0	1	2	3	4

5. Did the virtual white board help you understand what was happening during your ED stay?

Not at all	A little bit	Moderately	Quite a bit	Extremely
0	1	2	3	4

6. What did you think about the size of the virtual white board?
    - Too big
    - Adequately sized
    - Too small

7. Is there anything about your experience with the virtual white board that you think we should know? [BLANK TEXT BOX]

Part B: For all study participants

For the next series of statements use the following scale to describe your current stay in the emergency department (HCAHPS):

For the purpose of this survey, the term "care provider" includes physicians and physician assistants involved in your care.	
Were you seen by a care provider in a timely manner?	No, Yes somewhat, Yes mostly, Yes definitely
At the time of your arrival, did the registration staff treat you with courtesy and respect?	No, Yes somewhat, Yes mostly, Yes definitely
Were you kept informed about any delays?	No, Yes somewhat, Yes mostly, Yes definitely, Not applicable
Did the care providers listen carefully to you?	No, Yes somewhat, Yes mostly, Yes definitely
Did the care providers explain things in a way you could understand?	No, Yes somewhat, Yes mostly, Yes definitely
Did you know what to do if you had questions/concerns after discharge?	No, Yes somewhat, Yes mostly, Yes definitely, Not applicable

The next questions focus specifically on your experience with nurses during this visit.	
Did nurses treat you with courtesy and respect?	No, Yes somewhat, Yes mostly, Yes definitely
Did nurses listen carefully to you?	No, Yes somewhat, Yes mostly, Yes definitely
Did nurses explain things in a way you could understand?	No, Yes somewhat, Yes mostly, Yes definitely
Was there good communication between the different doctors and nurses?	No, Yes somewhat, Yes mostly, Yes definitely
Were you comfortable talking with nurses about your worries or concerns?	No, Yes somewhat, Yes mostly, Yes definitely
Did you have enough input or say in your care?	No, Yes somewhat, Yes mostly, Yes definitely

How likely would you be to recommend this facility to your family and friends?	Likely 0-10
