# Supplementary material for: A Pilot of Digital Whiteboards for Improving Patient Satisfaction in the Emergency Department: Nonrandomized Controlled Trial
Source: JMIR Form Res. 2023 Mar 21;7:e44725. doi: 10.2196/44725 (PMC10131606; doi:10.2196/44725)
Supplement: Multimedia Appendix 3 [file formative_v7i1e44725_app3.pdf]

Partners HealthCare System
Research Consent Form

Subject Identification

General Consent Form Template
Version Date: January 2019

Protocol Title: Improving patient satisfaction through information display using virtual whiteboards

Principal Investigator: Peter R Chai MD MMS

Site Principal Investigator: N/A

Description of Subject Population: Adult emergency department patients

About this consent form

Please read this form carefully. It tells you important information about a research study. A member of our research team will also talk to you about taking part in this research study. People who agree to take part in research studies are called "subjects." This term will be used throughout this consent form.

Partners HealthCare System is made up of Partners hospitals, health care providers, and researchers. In the rest of this consent form, we refer to the Partners system simply as "Partners."

If you decide to take part in this research study, you must sign this form to show that you want to take part. We will give you a signed copy of this form to keep.

Key Information

Taking part in this research study is up to you. You can decide not to take part. If you decide to take part now, you can change your mind and drop out later. Your decision won't change the medical care you get within Partners now or in the future.

The following key information is to help you decide whether or not to take part in this research study. We have included more details about the research in the Detailed Information section that follows the key information.

Why is this research study being done?

In this research study we want to learn more about how using a virtual whiteboard may help change mood and satisfaction with patients' emergency department (ED) experience.

How long will you take part in this research study?

If you decide to join this research study, it will take you about 24 hours, or as long as you are in the emergency department to complete the study. During this time, we will ask you to complete two surveys about the way you are feeling while you are in the emergency department.

What will happen if you take part in this research study?

If you decide to join this research study, the following things will happen:

- We will explain this consent form to you, and ensure you have time to think about your interest in participating in the study.
- You will need to sign this consent form.
- You will need to complete one survey at the start of the study where we will ask you about your experience with technology and your current mood.
- You will need to complete an exit surveys at the end of your stay to see if there has been a change in your mood or the way you feel about your emergency department stay.

Why might you choose to take part in this study?

We cannot promise any benefits to you from taking part in this research study. However, possible benefits may include an improved experience in the emergency department if you are in a room with a virtual whiteboard. Others who come to the emergency department in the future may benefit in the future from what we learn in this study.

Why might you choose NOT to take part in this study?

Taking part in this research study has some risks and requirements that you should consider carefully.

Important risks and possible discomforts to know about include the potential psychological discomfort of asking you to fill out surveys during the study. There is a risk of loss of confidentiality by having your personal health information displayed on a whiteboard for everyone in the room to see.

A detailed description of side effects, risks, and possible discomforts can be found later in this consent form in the section called "What are the risks and possible discomforts from being in this research study?"

Subject Identification

If you have questions or concerns about this research study, whom can you call?

You can call us with your questions or concerns. Our telephone numbers are listed below. Ask questions as often as you want.

Peter R Chai, MD, MMS is the person in charge of this research study. You can call him at 617-732-5640, Monday-Friday, 9:00 AM to 5:00 PM or email him at pchai@bwh.harvard.edu with questions. The BWH Emergency Department Research Team is available at 617-732-5638, Monday-Friday, 7:00 AM to 10:00 PM. You can also ask your nurse to page us at 11992 if you have questions regarding your study participation.

If you want to speak with someone **not** directly involved in this research study, please contact the Partners Human Research Committee office. You can call them at 857-282-1900.

You can talk to them about:

- Your rights as a research subject
- Your concerns about the research
- A complaint about the research
- Any pressure to take part in, or to continue in the research study

Detailed Information

A description of this clinical trial will be available on *http://www.ClinicalTrials.gov,* as required by U.S. Law. This Web site will not include information that can identify you. At most, the Web site will include a summary of the results. You can search this Web site at any time.

Why is this research study being done?

Keeping the patients in the emergency department (ED) aware of the care that they are getting is very important. The high number of patients in the ED sometimes causes long wait times and delays in giving these updates to patients. These delays may cause anxiety and impatience among patients and family members. The better patients are informed about their conditions, the less likely they will get anxious and the more satisfied they will be with their ED stay.

Many efforts have been done to improve patient-healthcare provider communications and satisfaction. For example, a dry erase whiteboard can provide basic information to patients- the names of their care team and plans for the day. However, this method is rarely, if ever, used given the additional time it takes to complete and update these boards by the busy care team.

Virtual whiteboards are one of the options to keep patients engaged with what their care team is planning for them. It can replace the traditional whiteboard that requires manual updating by a staff member. The screen can be designed to provide current information, such as room number, care team members information, pending procedures, treatment plans, important precaution, and laboratory results.

In this study, we will study the effects of virtual whiteboard screens on patients' satisfaction in the emergency department. One large screen will be attached securely on the wall several ED rooms

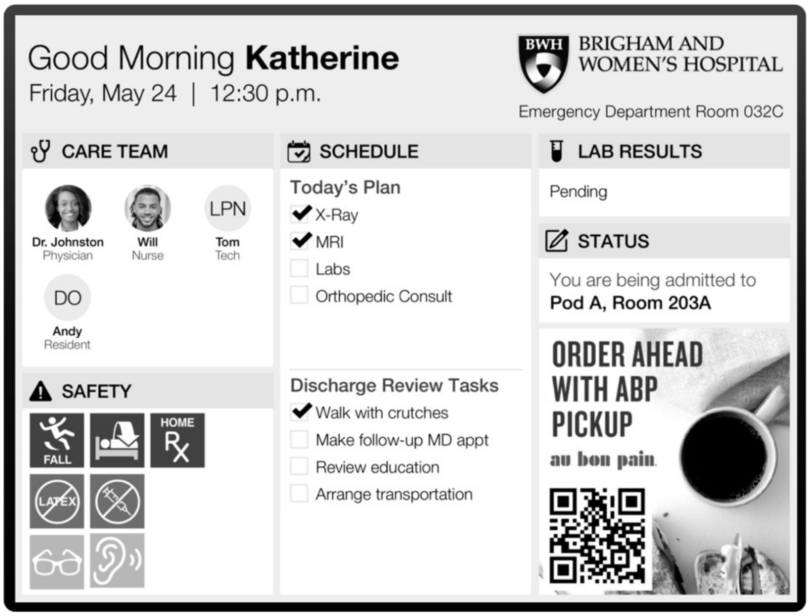

Above: example of the virtual whiteboard

Who will take part in this research?

We will be enrolling about 100 people in this study who are patients at Brigham and Women's Hospital Emergency Department. The sponsor, E-Ink holdings, is paying for this research to be done.

What will happen in this research study?

This study will take place only during your stay in the emergency department. There will be two groups in this study:

- Group 1: Patients who are triaged to a room in the emergency department with a virtual whiteboard.
- Group 2: Patients who are triaged to a room in the emergency department without a virtual whiteboard

Your assignment to these groups is based on availability of the rooms and your triage that is completed in the emergency department as part of your care by the clinical staff. If you are assigned to a room with a virtual whiteboard, you will be able to stay informed about your care in the ED.

If you agree to participate, we will send you the informed consent form to your email address and ask you to sign it electronically using Adobe Sign. You will only need an internet browser to complete this (no need to download additional apps) to your phone, tablet, or computer. If necessary, we may use a study device (a tablet) to obtain your signature. The study device will be cleaned before and after each use. We may also use a paper consent form as permitted.

Once you provide consent, we will administer a survey to you using either your own device or a study a tablet/computer. We will be asking questions about your experience using technologies like a smartphone and computer, your current mood state, your sense of security in the emergency department, and your orientation (e.g., the time and day of the week you think it is). We will also gather some information about you including the time you arrived in the emergency department, your length of stay, your initial chief complaint, your final diagnosis and medications administered to you. The information that we will collect from you or the medical record to be displayed on the virtual whiteboard will not be recorded for study purposes.

Once you complete this survey, your clinical care in the emergency department will continue. We will visit you when your clinical team has decided on a disposition for you (e.g., they have decided to admit, observe, discharge or transfer you). At that time, we will administer a second survey to you similar to the first one you did. In this survey, you will also be asked some questions about your emergency department stay.

If your emergency department visit is over 24 hours long, we will visit you at approximately 24 hours. At this visit, you will complete a survey around your mood, your orientation, and your satisfaction so far with your emergency stay.

How may we use and share your samples and health information for other research?

The information we collect in this study may help advance other research. If you join this study, we may remove all information that identifies you (for example, your name, medical record number, and date of birth) and use these de-identified data in other research. It won't be possible to link the information back to you. Information may be shared with investigators at our hospitals, at other academic institutions or at for-profit, commercial entities. You will not be asked to provide additional informed consent for these uses.

Will you get the results of this research study?

No. The research study we are doing is only a stepping-stone in understanding the use of a virtual whiteboard device in the emergency department. Therefore, no information about the results of this research study or the results of your individual participation in the research study

will be given to you or your doctor. The results of surveys you complete during the course of this study will not be given to your doctor or entered in your medical record.

What are the risks and possible discomforts from being in this research study?

The major discomfort associated with this study is the psychological discomfort associated with completing questionnaires. We will be asking you questions about your attitudes towards technology use, your current mood and your experience in the emergency department. You may feel some psychological distress associated with these questions. If you do experience these issues, you can notify one of our research assistants and you can skip questions that are upsetting to you. We additionally will not be collecting your name on the survey form, so there is no way for someone to link your name to the answers you have on these surveys.

There is a risk of disclosure of your private information during the study. While key information around consults ordered, imaging and labs planned and contact information of close contacts are already known to clinical staff, if you have visitors in the room, they may also view this information. We will keep the details to a minimum, for example, we will not display specific laboratory results, but instead only describe if the laboratory orders are pending, drawn, or resulted.

We will minimize the risk of possible infection by avoiding in-person interaction with you unless absolutely necessary. We will wear appropriate personal protective equipment based on our hospital infection control guideline when we are interacting with you.

What are the possible benefits from being in this research study?

There are no immediate benefits to you for participating in this study. If you are in a room with a virtual whiteboard, you may experience the benefit of having improved communication and satisfaction with the use of virtual whiteboard. This may happen because you will have more access to up-to-date information about your stay. Depending on the results of this study, we anticipate that virtual whiteboards like these could be used in other emergency departments or inpatient care units in the hospital.

What other treatments or procedures are available for your condition?

You can choose to not participate in this study. If you do so, you will still receive your usual care in the emergency department during this visit and any subsequent visits you may have.

Can you still get medical care within Partners if you don't take part in this research study, or if you stop taking part?

Yes. Your decision won't change the medical care you get within Partners now or in the future. There will be no penalty, and you won't lose any benefits you receive now or have a right to receive.

We will tell you if we learn new information that could make you change your mind about taking part in this research study.

What should you do if you want to stop taking part in the study?

If you take part in this research study, and want to drop out, you should tell us. We will make sure that you stop the study safely. We will also talk to you about follow-up care, if needed.

Also, it is possible that we will have to ask you to drop out of the study before you finish it. If this happens, we will tell you why. We will also help arrange other care for you, if needed.

Will you be paid to take part in this research study?

You will be given a $20 gift card for your participation in the study. You must complete all study procedures to receive the gift card.

What will you have to pay for if you take part in this research study?

Study funds will pay for certain study-related items and services. We may bill your health insurer for, among other things, routine items and services you would have received even if you did not take part in the research. You will be responsible for payment of any deductibles and co-payments required by your insurer for this routine care or other billed care. If you have any questions about costs to you that may result from taking part in the research, please speak with the study doctors and study staff. If necessary, we will arrange for you to speak with someone in Patient Financial Services about these costs.

What happens if you are injured as a result of taking part in this research study?

Injuries sometimes happen in research even when no one is at fault. There are no plans to pay you or give you other compensation for an injury, should one occur. However, you are not giving up any of your legal rights by signing this form.

If you think you have been injured or have experienced a medical problem as a result of taking part in this research study, tell the person in charge of this study as soon as possible. The researcher's name and phone number are listed in the beginning of this consent form.

If you take part in this research study, how will we protect your privacy?

Federal law requires Partners to protect the privacy of health information and related information that identifies you. We refer to this information as "identifiable information."

In this study, we may collect identifiable information about you from:

- Past, present, and future medical records
- Research procedures, including research office visits, tests, interviews, and questionnaires

Who may see, use, and share your identifiable information and why they may need to do so:

- Partners researchers and staff involved in this study
- The sponsor(s) of the study, and people or groups it hires to help perform this research or to audit the research
- Other researchers and medical centers that are part of this study
- The Partners ethics board or an ethics board outside Partners that oversees the research
- A group that oversees the data (study information) and safety of this study
- Non-research staff within Partners who need identifiable information to do their jobs, such as for treatment, payment (billing), or hospital operations (such as assessing the quality of care or research)
- People or groups that we hire to do certain work for us, such as data storage companies, accreditors, insurers, and lawyers
- Federal agencies (such as the U.S. Department of Health and Human Services (DHHS) and agencies within DHHS like the Food and Drug Administration, the National Institutes of Health, and the Office for Human Research Protections), state agencies, and

foreign government bodies that oversee, evaluate, and audit research, which may include inspection of your records

- Public health and safety authorities, if we learn information that could mean harm to you or others (such as to make required reports about communicable diseases or about child or elder abuse)
- Other: N/A

Some people or groups who get your identifiable information might not have to follow the same privacy rules that we follow and might use or share your identifiable information without your permission in ways that are not described in this form. For example, we understand that the sponsor of this study may use your identifiable information to perform additional research on various products or conditions, to obtain regulatory approval of its products, to propose new products, and to oversee and improve its products' performance. We share your identifiable information only when we must, and we ask anyone who receives it from us to take measures to protect your privacy. The sponsor has agreed that it will not contact you without your permission and will not use or share your identifiable information for any mailing or marketing list. However, once your identifiable information is shared outside Partners, we cannot control all the ways that others use or share it and cannot promise that it will remain private.

Because research is an ongoing process, we cannot give you an exact date when we will either destroy or stop using or sharing your identifiable information. Your permission to use and share your identifiable information does not expire.

The results of this research study may be published in a medical book or journal, or used to teach others. However, your name or other identifiable information **will not** be used for these purposes without your specific permission.

Your Privacy Rights

You have the right **not** to sign this form that allows us to use and share your identifiable information for research; however, if you don't sign it, you can't take part in this research study.

You have the right to withdraw your permission for us to use or share your identifiable information for this research study. If you want to withdraw your permission, you must notify the person in charge of this research study in writing. Once permission is withdrawn, you cannot continue to take part in the study.

If you withdraw your permission, we will not be able to take back information that has already been used or shared with others, and such information may continue to be used for certain purposes, such as to comply with the law or maintain the reliability of the study.

You have the right to see and get a copy of your identifiable information that is used or shared for treatment or for payment. To ask for this information, please contact the person in charge of this research study. You may only get such information after the research is finished.

Informed Consent and Authorization

Statement of Person Giving Informed Consent and Authorization

- I have read this consent form.
- This research study has been explained to me, including risks and possible benefits (if any), other possible treatments or procedures, and other important things about the study.
- I have had the opportunity to ask questions.
- I understand the information given to me.

Signature of Subject:

I give my consent to take part in this research study and agree to allow my health information to be used and shared as described above.

___________________________________ ____________ ____________
Subject Date Time (optional)

____
Printed Name

Subject Identification

Signature of Study Doctor or Person Obtaining Consent:

Statement of Study Doctor or Person Obtaining Consent

- I have explained the research to the study subject.
- I have answered all questions about this research study to the best of my ability.

Study Doctor or Person Obtaining Consent	Date	Time (optional)

Printed Name

Consent Form Version: 1.1
