# Supplementary material for: A Pilot of Digital Whiteboards for Improving Patient Satisfaction in the Emergency Department: Nonrandomized Controlled Trial
Source: JMIR Form Res. 2023 Mar 21;7:e44725. doi: 10.2196/44725 (PMC10131606; doi:10.2196/44725)
Supplement: Multimedia Appendix 4 [file formative_v7i1e44725_app4.pdf]

A Pilot of Digital Whiteboards for Improving Patient Satisfaction in the Emergency Department: Non-randomized controlled trial

Appendix

	Patients that did not complete the study protocol
Mean Age	53.7
Gender	60%M/40%F
Race:	*N*
White	7
Black	1
Asian	0
Other	2
Ethnicity:	*N*
Hispanic	2
Non-Hispanic	8
